# Decision-making in healthcare: a practical application of partial least square path modelling to coverage of newborn screening programmes

**DOI:** 10.1186/1472-6947-12-83

**Published:** 2012-08-02

**Authors:** Katharina E Fischer

**Affiliations:** 1 Helmholtz Zentrum München, German Research Center for Environmental Health, Institute for Health Economics and Health Care Management, Ingolstädter Landstr. 1, 85764, Neuherberg, Germany; 2 University of Hamburg, Hamburg Center for Health Economics, Esplanade 36, 20354, Hamburg, Germany

**Keywords:** PLS, Structural equation modelling, Quantitative research, Feasibility study, Model evaluation, Non-parametric, Fourth hurdle, Reimbursement, Neonatal, Europe

## Abstract

**Background:**

Decision-making in healthcare is complex. Research on coverage decision-making has focused on comparative studies for several countries, statistical analyses for single decision-makers, the decision outcome and appraisal criteria. Accounting for decision processes extends the complexity, as they are multidimensional and process elements need to be regarded as latent constructs (composites) that are not observed directly. The objective of this study was to present a practical application of partial least square path modelling (PLS-PM) to evaluate how it offers a method for empirical analysis of decision-making in healthcare.

**Methods:**

Empirical approaches that applied PLS-PM to decision-making in healthcare were identified through a systematic literature search. PLS-PM was used as an estimation technique for a structural equation model that specified hypotheses between the components of decision processes and the reasonableness of decision-making in terms of medical, economic and other ethical criteria. The model was estimated for a sample of 55 coverage decisions on the extension of newborn screening programmes in Europe. Results were evaluated by standard reliability and validity measures for PLS-PM.

**Results:**

After modification by dropping two indicators that showed poor measures in the measurement models’ quality assessment and were not meaningful for newborn screening, the structural equation model estimation produced plausible results. The presence of three influences was supported: the links between both stakeholder participation or transparency and the reasonableness of decision-making; and the effect of transparency on the degree of scientific rigour of assessment. Reliable and valid measurement models were obtained to describe the composites of ‘transparency’, ‘participation’, ‘scientific rigour’ and ‘reasonableness’.

**Conclusions:**

The structural equation model was among the first applications of PLS-PM to coverage decision-making. It allowed testing of hypotheses in situations where there are links between several non-observable constructs. PLS-PM was compatible in accounting for the complexity of coverage decisions to obtain a more realistic perspective for empirical analysis. The model specification can be used for hypothesis testing by using larger sample sizes and for data in the full domain of health technologies.

## Background

Across industrialized countries, many publicly funded healthcare systems have installed mechanisms to formally assess and appraise the coverage and reimbursement of health technologies [1]. Since the establishment of the UK National Institute for Health and Clinical Excellence (NICE) and similar institutions, research on healthcare decision-making has been extended beyond physician and patient behaviour [2]. Generally speaking, a decision produces an action as a result of the decision process. In healthcare, actions comprise provision of treatments, tests or clinical strategies [3]. In relation to coverage, a decision-making committee decides on reimbursement of a technology. Foremost is the decision process, which involves a variety of elements such as evidence assessment, stakeholder participation or application of appraisal criteria [4]. As such elements extend the complexity that comprise decisions and resulting actions, suitable methods are required for quantitative analysis.

Analogous to the regulation of environmental and safety issues [5], the implementation of formal coverage decision-making incurs benefits in terms of actions towards efficient allocation of resources. For example, the Swedish Pharmaceutical Benefits Board re-evaluated all antihypertensive treatments, which resulted in savings to the national health service of about 5% [6]. In contrast, third-party payers who intend to regulate coverage face costs because they need to enforce processes and conduct evidence assessments. From the manufacturers’ and patients’ perspectives, decision processes also delay market entry and the availability of treatments [7]. To understand the functioning of decision processes, instruments are needed to measure the consequences of different specifications of decision-making.

Coverage has been analysed by description of decision processes and qualitative and quantitative investigation of real-world decisions. Stafinski et al. provide an inventory of decision processes from 31 decision-makers based on available documentation [8]. Coverage decisions are subject to influences from policy, and complex technologies may challenge pre-defined processes. Thus, hypothetical description of decision procedures cannot capture such aspects. Vuorenkoski et al. identify six qualitative studies that focus on the description of selected aspects of past decision processes by a single committee [9]. Although qualitative approaches provide an opportunity to describe complex interrelationships between elements of decision-making in detail, they do not allow measurement of the strength of such effects mathematically. Using regression analysis, several studies have enforced quantitative approaches to analyse real-world decision-making [10-13]. These studies examine the relation between decision outcome and selected appraisal criteria. Furthermore, they focus on the UK NICE and the Australian Pharmaceutical Benefits Advisory Committee. A number of comparative studies apply descriptive methods but also concentrate on selected aspects, e.g. decision-making for cancer drugs [14-16].

To account for the complexity of decision-making, three aspects need consideration in empirical analysis: (1) Not all elements of decision processes can be measured by specification of variables that can be observed directly. For example, there is discussion about the legitimacy and transparency of decision processes being critical factors that support the appraisal stage [17]. Such concepts cannot be measured by observation of a single variable. So-called latent constructs or, composites are frequently defined from normative concepts and need operationalization by several observable indicators. (2) The network of linkages between elements of decision processes needs to be accounted for because the components of decision-making are not independent but influence each other. For example, Erntoft argues that cost-effectiveness considerations influence both assessment and appraisal [18]. (3) Multiple interrelationships further imply accurate specification of causal inferences. Correct description of cause and effect offers the opportunity to conduct empirical analysis from a more realistic point of view.

Structural equation modelling comprises a group of estimation algorithms that account for the complexity of coverage decisions. Linkages between several composites can be tested in a structural equation model (SEM) by a combination of factor and multiple regression analysis. Partial least square path modelling (PLS-PM) is a technique of structural equation modelling where the share of the variance explained for one or several endogenous constructs specified in the SEM is maximized through a series of ordinary least squares regressions [19]. PLS-PM is used by a growing number of research disciplines, e.g. strategic management or marketing [20]. However, its current application to decision-making in healthcare and, especially, to coverage is unclear.

The objective of this study is to present a practical application of PLS-PM to coverage decision-making and show how it offers a method for empirical analysis of situations that involve multiple interrelationships among several composites. This aim is achieved by identification of studies that have applied PLS-PM in the context of healthcare decision-making. The technique is then applied to a SEM that specifies a set of hypotheses on links between the components of decision-making. It is tested and evaluated for decisions to expand newborn screening programmes across Europe.

## Methods

### Identification of PLS-PM applications to decision-making in healthcare

Four databases were searched systematically to identify approaches that have applied PLS-PM to decision-making in healthcare and draw from existing specifications of structural models. Studies were included if they analysed decision-making in healthcare, at both individual and institutional level, including coverage and reimbursement. A detailed description of the study identification is presented in Additional file 1: Systematic search. The search identified two publications but none of them dealt with coverage decision-making. Downey and Sharp examined the adoption behaviour of worksite health promotion by company managers using the theory of planned behaviour [21]. Walter and Lopez examined the acceptance of information technologies in the medical sector assuming that physicians’ beliefs in their professional autonomy may be limited through IT [22]. Also, no studies that used covariance-based SEM to analyse coverage decisions were identified.

### Partial least square path modelling

Structural equation modelling includes several elements that are different from multivariate regression analysis [20]. A SEM consists of composites that are interrelated. Endogenous constructs depend on one or several exogenous constructs. In regression analysis, one dependent variable is explained by several independent variables. Specification of several endogenous constructs is not possible. In the structural model, links between several constructs can be defined. In the measurement models, the relation between a composite is defined by one or several manifest indicators that are observed directly in the data. Depending on the causality between construct and indicators, measurement models are either formative or reflective. In the former, the causality goes from the indicator to the construct, whereas for the latter, the direction of causality is reversed. Through application of an iterative procedure, the PLS-PM algorithm calculates the path coefficients between the composites and the scores of the constructs in the structural model and the weights and loadings of the manifest indicators in the measurement models in a sequence of ordinary least square regressions [19]. The software SmartPLS 2.0 M3 was used for estimation of the case study using the path weighting scheme to calculate the inner weights [23]. The mean replacement algorithm was selected to substitute missing values.

### The case study

The applicability of PLS-PM to coverage was assessed for a newly developed SEM which describes specific components of decision processes and their interrelationships. The motivation was to measure the effects between components that have been discussed in the literature separately but not in combination. For specification of the components which were regarded as composites, a set of indicators that has been developed to describe and structure the steps of coverage decision-making was used [4,24]. For the test of applicability, a data set of 55 coverage decisions made on newborn screening (NBS) technologies was available. Because of the small sample size and the expectation that decisions on NBS incur several peculiarities within the range of health technologies, it was not a suitable strategy to build the measurement models by use of clustering techniques [25,26]. Instead, the aim was to assess the SEM for hypotheses testing using larger sample sizes and in the full domain of health technologies. In the following, the components, the hypotheses, the empirical operationalization and the data are described.

#### Components of coverage decision-making and their interrelationships

A set of hypotheses was stated that rested on statements made in the literature, empirical observations and the logical combination of the components of decision processes. It was aimed to explore and predict the linkages to obtain a general perspective on coverage. Empirical studies have examined aspects that are frequently very specific for the decision-makers under consideration and, thus, their transferability is limited.

As no single theoretical framework exists that combines the components of decision processes, this model rested on two normative deliberations. To obtain reasonable and fair decision-making, the principles of procedural justices state that criteria have to be met such as transparency of and stakeholder participation in decision processes are ensured [4,17,27]. Besides, it is frequently argued that there is not a one single criterion for making reasonable decisions. Instead, a combination of medical, economic and other ethical criteria is used frequently [10-12,28-32]. What is unclear is the influence of the principles of procedural justice on the reasonableness of coverage decisions in terms of the criteria considered for appraisal.

Additionally, accounting for the use of assessment methods to gather evidence on the considered technology, the model specification thus consisted of four components: ‘transparency’, ‘participation’, ‘scientific rigour (of assessment)’ and ‘reasonableness’ [33-36]. A detailed description is provided in Table 1. As the appraisal of a technology is the final stage before the decision outcome is settled, it was hypothesized that the component ‘reasonableness’ is influenced by the others. It was further stated that both transparency and the degree of participation influence the rigour of assessment. An overview of the hypotheses that describe the links between the components is provided in Table 2.

**Table 1:** Specification of constructs and measurement models for SEM of coverage decision-making

**Construct**	**Construct description**	**Indicator**	**Indicator description**
Participation	Different stakeholder groups are involved at various stages of decision processes to ensure that their interests are not neglected [34,35,54,55].	Number of different types of participating stakeholders (i.e. service provider(s), payer, government, patients/patient representative(s), industry)	Degree of participation reflected by number of types of stakeholders involved in the decision process. High diversity of stakeholders increases the possibility that particular interests of single stakeholders are balanced out.
		Degree of stakeholder involvement (i.e. information provision, appeal, voting, one indicator per type of involvement)	Number of stakeholders involved at stages in decision process. More involvement opportunities result in stronger participation.
Transparency	Processes are considered transparent if relevant information is provided so that decisions can be retraced [36,56]. More transparency improves the extent to which a decision can be controlled. Transparency is reflected by the degree of detail in the documentation of processes and decision outcomes [33,56].	Amount of information published during or after decision process	Degree of transparency reflected by the amount of documents published for each decision.
		Type of information provided	Degree of transparency reflected by the diversity of published information provided – i.e. whether it relates to the process or decision outcome or both.
Scientific rigour of assessment	Scientific rigour is defined by the methodological standards for generating evidence. The assessment of effectiveness may range from collecting expert opinions to quantitative meta-analyses of studies. Assessment of costs may go from rough estimates to comprehensive cost-effectiveness or budget impact analyses. Rigorous assessments are prerequisites to reasonable decisions that are evidence based and accepted by informed people [36,56].	Scientific rigour in assessment of effectiveness	The degree of scientific rigour is positively reflected by the degree of methodological standards used for the assessment of effectiveness.
		Scientific rigour in assessment of costs/cost-effectiveness	The degree of scientific rigour is positively reflected by the degree of methodological standards used for the assessment of costs/cost-effectiveness.
Reasonableness	Reasonableness is defined as the extent to which typically accepted criteria are considered in technology appraisal [1,18,32]. The higher their relevance and the number of criteria considered, the stronger the degree of reasonableness is reflected.	Relevance of criteria that contribute to reasonable appraisal (i.e. *clinical* (effectiveness: health benefit; effectiveness: other benefit (e.g. knowledge of diagnostic test result)), *economic* (cost-effectiveness, budget impact) and *other ethical criteria* (severity of the disease, equitable access to care), one indicator per criterion)	The higher the relevance of clinical, economic or other ethical criteria, the higher the degree of reasonableness of the decision.

**Table 2:** Specification of hypotheses: links between components of coverage decision processes

**Component: cause**		**Component: effect**	**Hypothesized relationship**
Transparency	→	Reasonableness	The more documents are provided that strongly relate to dissemination of the process and decision outcome, the higher is the extent to which the decision is appraised against reasonable criteria because this facilitates a better control of the decision-makers.
Participation	→	Reasonableness	The more stakeholders participate in different stages of decision-making, the more they mutually ensure that the technology is appraised against reasonable criteria.
Scientific rigour of assessment	→	Reasonableness	The higher the methodological standards by which the technology is assessed, the higher is the extent to which the decision is appraised against reasonable criteria, because decision-makers can draw upon better evidence regarding whether the criteria are met.
Transparency	→	Scientific rigour of assessment	The more documents are provided that strongly relate to dissemination of the process and decision outcome, the higher the methodological standard of technology assessment because the methodological quality can be better controlled by the scientific community.
Participation	→	Scientific rigour of assessment	The more stakeholders participate in different stages of decision-making, the higher the scientific standard of technology assessment because more evidence is identified and improvements of a weak evidence basis can more easily be enforced.

#### Specification of the structural equation model

To specify the SEM, the components rested on an existing structuring and operationalization for empirical analysis. The structure outlined by Rogowski et al. captures both process and appraisal criteria [4]. It describes the stylized steps of a decision process from the point where a technology enters a healthcare market to its diffusion into routine use. The following steps were considered: stakeholder ‘participation’; ‘publication’, which reflects information about the transparency of decision processes; ‘scientific rigour of assessment’ by methods such as systematic literature review or cost-effectiveness analysis; and ‘appraisal’ in terms of decision-making criteria which determine whether the technology should be funded given the available evidence on effectiveness and costs and additional ethical considerations. This step reflects the reasonableness of decision-making. Each step in the framework corresponds with a set of indicators for empirical operationalization which has been proposed in the literature [24]. This collection of variables has been validated by a small number of decisions and expert discussions.

**Figure 1:**
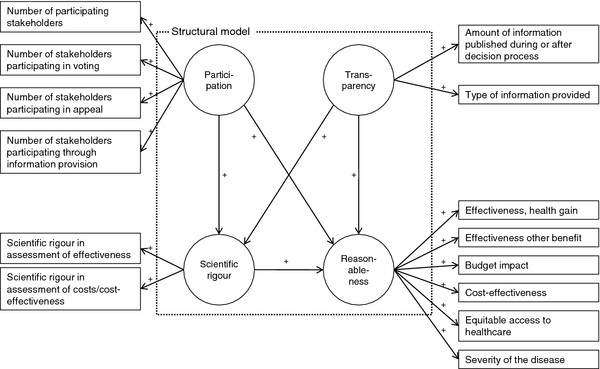
SEM for coverage decision-making.

Translating the framework into a SEM, the steps of decision-making were considered as composites that define the components, whereas the set of indicators provided the definition of the measurement models (Figure 1). A description of constructs and corresponding measurement models which were defined in the reflective mode is provided in Table 2.

#### Data

Data was obtained from an internet survey of decisions for expansion of NBS programmes in the European Union. NBS includes a number of promising technologies relevant to third-party payers. Of these, tandem mass spectrometry allows screening of multiple disorders in one step. Experts provided information about decisions made between 2005 and 2009 on screening for medium-chain acyl-CoA dehydrogenase deficiency, cystic fibrosis, congenital adrenal hyperplasia or other conditions. Forty-three respondents completed the questionnaire and the response rate was 70%. In the questionnaire, at least one question was stated for each construct (see Additional file 2: Survey questionnaire). A detailed description of the survey has been provided elsewhere [37].

From 21 countries, a total of 55 decisions were obtained. All variables to estimate the SEM arose from the data set and were specified as ordinal or count variables according to the definitions provided in Table 2. An overview of descriptive statistics including the frequencies of categories for each indicator is provided in Table 3. Data preparation was performed with SAS Version 9.2 [38].

**Table 3:** Frequencies of indicators for case study on NBS in Europe

**Construct/Indicator**	**Categories**		
**Construct: Participation**			
		Mean	St.d.
Number of stakeholders involved in decision process		2.84	1.23
Number of stakeholders participating through information provision		1.31	1.10
Number of stakeholders participating in appeal		0.31	0.60
Number of stakeholders participating in voting		1.13	0.98
**Construct: Transparency**			
		n	%
Type of information provided	0 - No information available	5	9.09
	1 - Only process-related information available	3	5.45
	2 - Only outcome-related information available	30	54.55
	3 - Outcome- and process related information available	17	30.91
	4 - Full documentation	0	0.00
		Mean	St.d.
Amount of information published during or after decision process		2.05	1.39
**Construct: Scientific rigour of assessment**			
		n	%
Scientific rigour in assessment of effectiveness	0 - No assessment of effectiveness/other	1	1.82
	1 - At least based on expert opinion	10	18.18
	2 - At least systematic literature review	36	65.45
	3 - At least quantitative meta-analysis of studies	8	14.55
Scientific rigour in assessment of costs/cost-effectiveness	Missing	4	7.27
	0 - No assessment of costs/CE	2	3.64
	1 - Cost estimate	39	70.91
	2 - Cost-effectiveness analyses	10	18.18
**Construct: Reasonableness**			
Aspects considered for appraisal		n	%
Effectiveness, health gain	0 - Not relevant	6	10.91
	1 - Relevant	14	25.45
	2 - Strongly relevant	35	63.64
Effectiveness, other benefit	0 - Not relevant	38	69.09
	1 - Relevant	14	25.45
	2 - Strongly relevant	3	5.45
Budget impact	0 - Not relevant	38	69.09
	1 - Relevant	15	27.27
	2 - Strongly relevant	2	3.64
Cost-effectiveness	0 - Not relevant	36	65.45
	1 - Relevant	12	21.82
	2 - Strongly relevant	7	12.73
Effect on equitable access to healthcare	0 - Not relevant	32	58.18
	1 - Relevant	22	40.00
	2 - Strongly relevant	1	1.82
Severity of the disease	0 - Not relevant	12	21.82
	1 - Relevant	15	27.27
	2 - Strongly relevant	28	50.91

### Evaluation of the structural equation model

For PLS-PM, no global goodness-of-fit criterion exists because it is assumed that the variance is distribution free. Alternatively, a set of standard measures for PLS-PM exists according to which reliability and validity of the model estimation were evaluated [19,20,39]. Although all results were obtained from the iterative estimation at one time, the reflective measurement models were evaluated before the structural model was assessed by another set of measures. Reliable and valid measurement models are prerequisites for the evaluation of the structural model. All measures were obtained from the reports provided in SmartPLS [23]. As the SEM does not contain formative measurement models, these measures were neglected.

#### Evaluation of reflective measurement models

The reliability of the reflective − i.e. the causality goes from the construct to the indicators − measurement models was assessed at construct and indicator level. At construct level, composite reliability was considered, that measures whether the indicators consistently represent the same construct and the systematic error is considered to be zero. Composite reliability accounts for the indicators’ weights and is considered acceptable above a value of 0.7 for established constructs and above 0.6 in the early stages of research [19]. At indicator level, the factor loadings reflect the indicator’s variance explained by the construct. To assume reliable measures, at least 50% of the variance should be explained, which is reflected by loadings greater than $0.7\approx \sqrt{0.5}$.

Validity was evaluated through convergent and discriminant validity. Convergent validity assumes that the set of indicators uniquely represents the underlying construct. For this purpose, the average variance extracted (AVE) was considered to measure the variance of the indicators of the reflective construct relative to the total amount of variance, including the variance of the measurement error. To evaluate discriminant validity, two criteria were used that appraise whether the reflective constructs are sufficiently distinct from each other. First, the Fornell–Larcker criterion compares the AVE of a composite with the squared correlations between the construct and any other construct of the model. Discriminant validity can be stated if the AVE of the composite is larger than any other squared correlation. Second, the cross-loadings were compared to see whether the loadings with the corresponding construct were the highest.

#### Evaluation of the structural model

At the level of the structural model, the path coefficients were evaluated first in terms of sign and significance. They reflect the standardized beta coefficients for which asymptotic t-statistics were computed from the bootstrapping procedure. Second, the determination coefficient *R*^*2*^ – analogous to multiple regression – reflects the level or share of the composites’ explained variance. It was analysed for the endogenous composites. Third, the effect size *f*^*2*^ was computed to determine whether an exogenous construct substantially influenced an endogenous construct. Similar to the traditional partial F-test, the change in *R*^*2*^ is computed if the respective exogenous construct is omitted. Fourth, the predictive relevance of the structural model was evaluated to determine how well the model parameters can be reconstructed using the model and the PLS parameters. For this purpose, the blindfolding procedure was performed in SmartPLS, which calculates Stone–Geisser’s *Q*^*2*^ (omission distance: 7). It displays the relative predictive impact of a construct. Values above zero indicate the presence of predictive relevance [40].

### Model selection

As the SEM is at an early stage of development, it was assessed whether improvements in respect of model reliability and validity can be achieved. The model selection was based on the evaluation measures of the measurement models. It was suspected that, compared with other technologies, the case study of NBS inherits some peculiarities. Cost-effectiveness as decision criterion may have not been as relevant as for pharmaceuticals [41]. Besides, considered conditions are typically severe heritable diseases which increase mortality and morbidity from infant age [42]. A modified SEM was then estimated and evaluated. Besides obtaining first estimations of the hypotheses for the case study of NBS, it was aimed to discuss implications for a general application of the SEM and PLS-PM as modelling technique for coverage decision-making.

## Results

### Results for reflective measurement models

**Figure 2:**
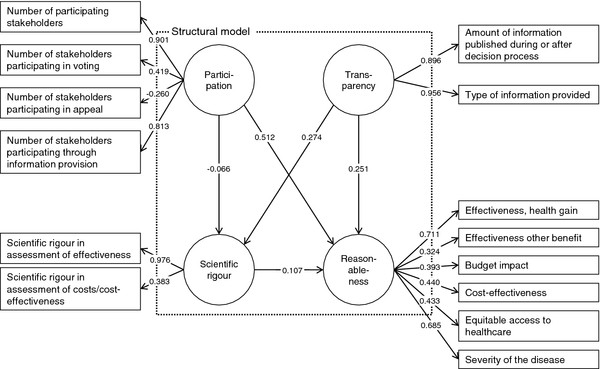
**SEM, estimation results after first estimation.**1

In the first estimation, the evaluation measures of reflective measurement models displayed some weaknesses. Figure 2 displays the estimation results. A detailed overview of all evaluation measures is provided in Additional file 3: Evaluation measures. Reliability at construct level assesses whether the indicators consistently represent the same construct and the relevance of systematic errors. Accounting for the stage of development of the SEM, this was fulfilled for all constructs. Values of composite reliability were at least 0.6, which is acceptable at early stages of research [19].

Regarding indicator reliability, the factor loading, which represents the variance explained by the construct, was below 0.7 in five of six indicators of the ‘reasonableness’ construct. This indicates a lack of reliability as the shared variance between the construct and the indicators is then lower than the variance of the measurement error. Below a value of 0.4, indicators should be eliminated [43]. The indicators ‘number of stakeholders participating in appeal’ of the ‘participation’ construct and the ‘relevance of effectiveness in terms of other benefits in the ‘reasonableness’ construct were the indicators with values clearly below 0.4.

Regarding validity, the evaluation measures for convergent and discriminant validity also indicated a need for model modification. The average variance extracted (AVE), which describes whether the set of indicators uniquely represents the underlying construct, was less than 0.5 for the constructs ‘participation’ and ‘reasonableness’. This indicates that more than half the variance extracted results from the variance of the measurement error. Concerning discriminant validity to assess whether the constructs are sufficiently distinct from each other, the evaluation measures disclosed weaknesses in the ‘participation’ and ‘reasonableness’ constructs. The Fornell−Larcker criterion, which compares the AVE of a composite with the squared correlations between the construct and any other construct, revealed that at least one squared correlation with another construct was higher than the AVE for the construct ‘reasonableness’. A comparison of the loadings with the indicators’ cross-loadings with other constructs showed that values were at a maximum for all indicators except ‘number of stakeholders participating in appeal’ of the ‘participation’ construct.

According to identified weaknesses, two indicators were removed because of their lack of reliability. Concerning the construct ‘reasonableness’, the indicator that reflects the relevance of effectiveness in terms of other benefits was removed. The same applied for the indicator ‘number of stakeholders participating in appeal’ of the ‘participation’ construct.

**Figure 3:**
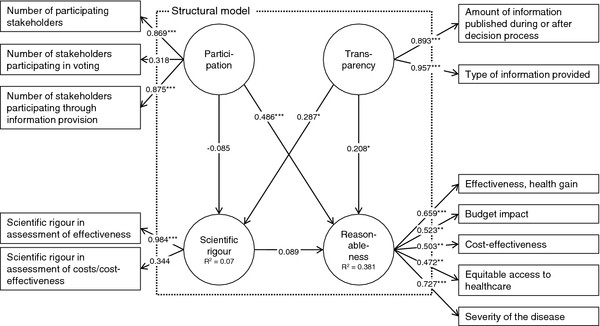
**Selected, estimation results for newborn screening programmes in Europe.** legend: ***: p-value < 0.01; **: p-value < 0.05; *: p-value < 0.1.

After re-estimation, criteria for reliability were fulfilled at the construct and indicator level for the reflective measurement models. The selected SEM is depicted in Figure 3, which includes the estimation results. Regarding reliability at indicator level, all factor loadings were higher than 0.4 except for the indicators ‘number of stakeholders involved in voting’ (loading 0.318) and ‘scientific rigour in assessment of effectiveness’ (loading 0.344). Convergent validity was fulfilled for all constructs except the construct ‘reasonableness’. Here, the AVE has increased to a value of 0.342 but was still below 0.5. Discriminant validity could be stated without caveat for all constructs. Both the Fornell−Larcker criterion was fulfilled and indicator loadings were at maximum at the assigned constructs. Bearing the weaknesses of indicator reliability and convergent validity in mind, the measurement models were considered acceptable for evaluation of the structural model.

### Results for the structural model

The structural model was not rejected. The measures for *R*^*2*^ revealed that, for the construct ‘reasonableness’, 56% of the share of the variance and, for the construct ‘scientific rigour’, 7% of the share of the variance was explained in the model. Three of the five hypotheses were supported for decision-making on NBS technologies. Their path coefficients, which are interpreted as standardized beta coefficients, had the hypothesized direction and were significant at least at the 90%- level. A higher degree of stakeholder participation (path coefficient 0.486; p < 0.001) and transparency (0.208; p = 0.098) increased the degree of making reasonable coverage decisions. Furthermore, the degree of transparency positively influenced the degree of scientific rigour (0.287; p = 0.051). The link between the constructs ‘scientific rigour’ and ‘reasonableness’ had the intended sign but was not significant (p = 0.57). It also had a very small path coefficient. A link between participation and ‘scientific rigour’ was not supported, as the path was negative and not significant (p = 0.58). Thus, the degree of participation did not influence the rigour of assessment.

The values of the effect size *f*^*2*^ which reflect the influence of the exogenous on the endogenous constructs in terms of the share of the variance, showed that the constructs ‘participation’ and transparency’ contributed to explanation of the endogenous constructs to varying degrees. For the construct ‘participation, no effect was found for the contribution to the construct ‘scientific rigour’ while a medium effect was identified for the contribution to the construct ‘reasonableness’. The construct ‘transparency’ both contributed to explanation of the endogenous constructs with a weak effect. The Stone−Geisser test, which assesses the model’s capability to predict, revealed mixed results. No predictive relevance was identified for the construct ‘scientific rigour’ (*q*^*2*^=−0.01) while a small predictive relevance was found for the reasonableness construct (*q*^*2*^ = 0.04).

## Discussion

The applicability of PLS-PM to coverage needs critical reflection as comparison with existing studies is not possible. The stated constructs can be used for further investigation of linkages between the components of coverage decision-making. The measurement models could be operationalized in a reliable and valid manner. Although the supposed links between observable indicators and constructs could be validated at measurement model level, further exploration is needed at the level of the structural model, especially if the tested hypotheses hold for other technologies. In spite of this, the qualitative interpretations of the results provide insight into whether PLS-PM produces plausible estimates and whether it is a suitable application for hypothesis testing using large data sets.

The case study reveals that the influence from the degree of stakeholder participation on reasonableness is about twice as influential as the degree of transparency in European NBS decisions. Besides, the degree of transparency significantly influences the level of methodological standards for evidence assessments. Thus, the estimation results are capable of demonstrating that the process components of coverage decisions that describe elements of procedural justice and definition of substantive appraisal criteria influence each other. No influence was found for the path between participation and scientific rigour which suggests that NBS technologies were assessed independently from the influence of stakeholders. Also, the *R*^*2*^ for the construct ‘scientific rigour’ was small which suggests that it is not well explained by the exogenous constructs of the SEM. On the contrary, considering the multiplicity of influences on coverage decision-making, the value of *R*^*2*^ of the ‘reasonableness’ construct can be considered acceptable. Examples are institutional configurations such as the level of decision-making or the implementation of the technology within the reimbursement scheme which are not described in this model [44].

Some features of NBS in comparison with other health technologies need to be kept in mind when interpreting the results. Although evaluation measures for the construct ‘scientific rigour’ are acceptable, the path coefficient had no significant influence on the degree of ‘reasonableness’. This finding is supported in the literature on evaluation of NBS technologies, which states that cost-effectiveness information have frequently not been considered for appraisal [41]. This is also in line with the small correlation between the indicator that reflects the scientific rigour of assessing costs/cost-effectiveness and the construct. Compared with technologies such as pharmaceuticals, coverage may not have been regulated as strictly, which is indicated by a low uptake of health technology assessment [37]. Furthermore, survey respondents stated that funding of the screening tests was frequently negotiated between the payer and service providers, for which processes have not been defined (yet) or did not require disclosure of information. Thus, the degree of stakeholder participation had the strongest influence of the reasonableness of decision-making and was significant. Regarding the construct ‘reasonableness’, the effectiveness in terms of the health gain from testing and the severity of the disease were the indicators that reliably reflected the construct in the selected model estimation. A reason why cost-related aspects were not as meaningful may be the relatively low cost of the screening technologies and, for selected disorders, the high perceived effects from screening of newborns [42]. Decisions on NBS were often made by institutions that do not typically decide on coverage of health technologies. Especially for pharmaceuticals, criteria such as cost-effectiveness and budget impact are perceived as being more relevant [1,13]. Thus, for the construct ‘reasonableness’, all criteria should be used at the start of the analysis if decision-making on health technologies is examined. Also, principal component or factor analysis on all observed indicators could be applied if the sample size is sufficient.

PLS-PM manages to account for the complexity between the components stated in the model. As the goal of PLS-PM is to support the exploration and prediction of models under development, it provides guidance as to which link suggested for coverage decisions can be identified empirically. Before the other hypotheses are ultimately rejected, further evaluation is needed about whether this is also true for other technological areas. The relation between scientific rigour and reasonableness might be significant as these components are tied more closely in other processes, e.g. the technology appraisal by the UK NICE [45]. Similarly, the relation between the degree of participation and scientific rigour might be meaningful in other technological domains. In many countries, pharmaceutical manufacturers need to submit evidence on their products to obtain coverage [46]. Typically, this was not the case for NBS technologies. Finally, the model demonstrated that PLS-PM may be applicable for contexts of decision-making where ‘soft’ influences with high complexity and multiple links matter. Besides decision-analytic modelling, such approaches are demanded in healthcare, e.g. shared decisions between patients and physicians [47]. Nevertheless, a correct specification of the theoretical model is a crucial requirement to accurately interpret the empirical results. The conceptual specification of this SEM needs further elaboration through expert validation and discussion of the theoretical foundations. Furthermore, making confirmatory statements is limited when using PLS-PM. Instead, covariance-based SEM should be used [20].

The test of applicability of PLS-PM has some limitations. The estimation was based on a small sample and on decisions made for a very specific technological area. However, the sample size was sufficient according to established rules of thumb for PLS-PM [20]. The PLS-PM results were not compared with other modelling techniques such as covariance-based SEM or multivariate regression analysis. However, application of these techniques is limited for the reasons for which PLS-PM was considered suitable, namely the capability to account for small sample sizes and no possibility of defining formative measurement models. Omitting possible influences of the survey sample, it was not possible to split the data for the different stages of model development (i.e. model specification, test of significance) because of the small sample size. A split sample design is appropriate for this purpose but was not applied.

Regarding model specification, theory dependency of the results cannot be neglected because the causal dependencies were specified without testing for other possible structures for the network of considered components. Bayesian network analysis would be suited to train and validate the model structure [48,49]. However, required data was missing for this purpose. No expert opinion about the possible causal relationships and no information about the probabilistic relationships between constructs and indicators were available. By collection of further information, e.g. through an expert workshop, the model estimation could be used in future studies for validation. However, while theoretical considerations are developing, this study provides a first exploratory estimation of a SEM for coverage decision-making as well as measurement models that can be used for further analysis.

Potential unobserved heterogeneity between decisions has not been accounted for. Decision practices may differ by healthcare system or technological characteristics. However, no distinct explanatory variables have been suggested for coverage decision-making in the literature. To treat heterogeneity, methods for PLS-PM are available to identify plausible clusters ex post. Such techniques have been proved appropriate in marketing research [50], and similar approaches have been used in other contexts of health economics [51].

This study has quantitatively assessed the procedural aspects of decision-making such as stakeholder participation and transparency, which have been claimed as relevant for fair and legitimate decision-making [17]. The accountability for reasonableness framework has predominantly been evaluated by qualitative approaches for which the evaluation of the effects frequently remains subject to judgements from a few case studies [52]. Furthermore, the framework neglects appraisal criteria and consensus on adequate assessment methods [53]. Specification of composites and several endogenous variables allows the combining of both process and appraisal simultaneously.

Compared with existing empirical research, the application of PLS-PM demonstrates that dependencies between several constructs can be tested when using small sample sizes. Previous work focuses on dependencies between the decision outcome and selected appraisal criteria [10-13]. Relating to the work of Vuorenkoski et al., the estimation results have reconfirmed the relevance of transparency and stakeholder participation to ensure the quality of decision-making in the case of NBS [9].

## Conclusions

This study presents a practical application of PLS-PM to a set of hypotheses for coverage decision-making on newborn screening programmes. Although PLS-PM is established in areas such as marketing and a comprehensive set of evaluation measures is available to assess model reliability and validity, the SEM on coverage in this study is among its early applications in healthcare. Accounting for the early stage in research, the estimates produce measurement models for the constructs ‘transparency’, ‘participation’, ‘scientific rigour’ and ‘reasonableness’, which can be used for further model validation and hypothesis testing. The structural model results support the presence of three influences for decisions on newborn screening in Europe: (1) the influence of stakeholder participation and (2) transparency on the degree of making reasonable coverage decisions; and (3) the effect of transparency on the degree of scientific rigour of assessment.

PLS-PM allows the testing of hypotheses in situations where there are multidimensional interrelationships and composites that need operationalization by several observable indicators. This estimation technique is thus compatible in accounting for the complexity of coverage decision-making to obtain a more realistic perspective of the influences between components of decision processes and appraisal criteria.

## Abbreviations

AVE, Average variance extracted; NBS, Newborn screening; PLS-PM, Partial least square path modelling; SEM, Structural equation model.

## Competing interests

This research was carried out on behalf of the Helmholtz Zentrum München, German Research Center for Environmental Health (HMGU). The HMGU is an independent organization funded by the German and Bavarian governments. During the course of this study, the author was an employee of the HMGU and neither did nor does have a conflict of interest with regard to this study.

## Author’s contribution

KF was fully responsible for all activities involved including the development of the research design, the model estimation and evaluation and drafting of the manuscript.

## Pre-publication history

The pre-publication history for this paper can be accessed here:

http://www.biomedcentral.com/1472-6947/12/83/prepub

## Supplementary Material

Additional file 1 Systematic search. Click here for file

Additional file 2 Survey questionnaire. Click here for file

Additional file 3 Evaluation measures. Click here for file
