# Supplementary material for: Decision-making in healthcare: a practical application of partial least square path modelling to coverage of newborn screening programmes
Source: BMC Med Inform Decis Mak. 2012 Aug 2;12:83. doi: 10.1186/1472-6947-12-83 (PMC3444310; doi:10.1186/1472-6947-12-83)
Supplement: Additional file 1 — Systematic search. [file 1472-6947-12-83-S1.doc]

# Decision-making in healthcare: a practical application of partial least square path modelling to coverage of newborn screening programmes

## Additional File 1: Description of systematic search to identify studies that apply PLS-PM to decision-making in health care

***Inclusion criteria***

- Healthcare application
- PLS-PM estimation technique for structural models
- Articles published after 1980
- Language: English, French or German

***Searches in databases***

Databases: Business Source Complete and EconLit (via EBSCOhost), searched 4 November 2011

| **Search #** | **Search terms** | **Results** |
| --- | --- | --- |
| S1 | structural equation model* or structural model* or partial least square or path model* | 17,036 |
| S2 | decision or decision*making | 544,347 |
| S3 | health or medicine or care | 849,326 |
| S4 | (S1 and S2 and S3), Limiters - Published Date from: 19800101-20111231 | 180 |

Databases: EMBASE and MEDLINE (via OvidSP), searched 4 November 2011

| **Search #** | **Search terms** | **Results** |
| --- | --- | --- |
| 1 | structural equation model*.ti,ab. | 10,348 |
| 2 | structural model*.ti,ab. | 13,092 |
| 3 | partial least square*.ti,ab. | 8,395 |
| 4 | PLS | 8,834 |
| 5 | path model*.ti,ab. | 1,341 |
| 6 | decision.ti,ab. | 249,052 |
| 7 | decision*making.ti,ab. | 1,288 |
| 8 | 6 or 7 | 250,016 |
| 9 | 1 or 2 or 3 or 4 or 5 | 36,491 |
| 10 | 8 and 9 | 378 |
| 11 | limit 10 to (yr="1980 - 2011" and (english or french or german)) | 372 |
| 12 | remove duplicates from 11 | 209 |

**Selection of studies**

Studies were first screened by title and abstract and then screened in full text. References without an abstract, theses and conference contributions were excluded.

Records identified through database searching

(n =387)

Records after duplicates removed
(n = 375)

Records screened
(n = 375)

Records excluded
(n = 350)

Full-text articles assessed for eligibility
(n = 25)

Full-text articles excluded, with reasons
(n = 23)

Number of studies applying PLS-PM
(n = 2)
