# Supplementary material for: Decision-making in healthcare: a practical application of partial least square path modelling to coverage of newborn screening programmes
Source: BMC Med Inform Decis Mak. 2012 Aug 2;12:83. doi: 10.1186/1472-6947-12-83 (PMC3444310; doi:10.1186/1472-6947-12-83)
Supplement: Additional file 2 — Survey questionnaire. [file 1472-6947-12-83-S2.pdf]

Decision-making in health care: A practical application of partial least square path modelling to coverage of newborn screening programs

Additional File 2: Survey questionnaire

HelmholtzZentrum münchen
German Research Center for Environmental Health

Institute of Health Economics

and Health Care Management

HIScreenDiag - Building a Tool to Evaluate and Improve Health Investments in Screening and Diagnosis of Disease

We would like to understand how coverage and reimbursement decisions about genetic tests are made in your country. The majority of the questions are structured. Please try to answer every question. If you feel that the responses offered do not closely match how decisions are made in your country, then please feel free to add some further information in the space provided. If you have any questions or queries about this survey, please contact:

Katharina Fischer
Email: katharina.fischer@helmholtz-muenchen.de
Telephone: +49-89-3187-4157

All provided information is treated confidentially and will only be used for scientific and non-commercial purposes.

Please return this questionnaire by mail to:

Helmholtz Zentrum Muenchen
Institute of Health Economics and Health Care Management
Katharina Fischer
Ingolstaedter Landstr. 1
85764 Neuherberg/Germany

Or fax to:

+49 89 3187-3375

To better guide you through the survey, we provide definitions and two examples of completed decision processes. The purpose of these examples is to help you understand how to complete the survey. They are provided in footnotes at the end of each page. The examples are:

1.) In July 2006, the UK National Screening Committee decided to introduce provision and funding of neonatal screening of sickle-cell disorders by the English National Health Service.

2.) In 1998, the Swedish regions of Scania, Kronoberg, Halland and Blekinge decided to jointly offer counselling and screening of families with a family member having hereditary non-polyposis colorectal cancer (HNPCC). The coverage decision was last updated in May 2006.

In the following questions, we will ask you about coverage/reimbursement decision options on screening and diagnosis with a specific focus on genetic testing technologies.

**1.**	**Please name those coverage/reimbursement decision options for which you are familiar with past decision making in your country (between 2004 and 2009). If no decisions were made on the options provided for newborn screening, you may insert any other option from the area of genetic testing where a decision was made in your country.** [1]
	Multiple answers are possible.<br><br>Newborn screening<br><br>Please specify: ____________________<br><br>Please specify: ____________________<br><br>Please specify: ____________________<br><br>Further decision options in the area of genetic testing and screening<br><br>Please specify: ____________________<br><br>Please specify: ____________________<br><br>Please specify: ____________________
	**Please use this space here to provide further information. For example, you can use this space to explain why the most appropriate answer is not among the options or if the question is not applicable to your country. In case you don't know the answer right away we would be grateful about a hint to further information.**

[1] **Coverage/reimbursement:** (Partial) funding of any medical service provision (e.g. genetic testing in population-based screening programs) by a third party payer being either public or private
Decision option: A technology (e.g. pharmaceutical, medical procedure, diagnostic service) to prevent or treat a condition or disease which a third party payer can choose to cover/reimburse
Genetic test: The analysis of human DNA, RNA, chromosomes, proteins, and certain metabolites in order to detect heritable disease-related genotypes, mutations, phenotypes, or karyotypes for clinical purposes. Such purposes include predicting risk of disease, identifying carriers, establishing prenatal and clinical diagnosis or prognosis. Prenatal, newborn, and carrier screening, as well as testing in high risk families, are included (Task Force on Genetic Testing, 1997).

IF YOU COULD NOT SPECIFY ANY DECISION OPTION, GO TO QUESTION 1b, OTHERWISE CONTINUE!

1a.	**Please insert a maximum of three decision options from those you stated in the first question for further evaluation. Throughout the questionnaire, the numbers (1), (2) and (3) will correspond with the decision options you stated here.**
	If you have selected less than three decision options, please select all for further evaluation.
	(1)
	(2)
	(3)

ONLY ANSWER THE QUESTIONS ON THIS PAGE, IF YOU DID NOT SPECIFY ANY DECISION OPTIONS IN QUESTION 1.

1b.	**Can you provide information why no coverage/reimbursement decision option has been selected for your country?**

1c. Are you aware of any other person who might be able to provide information on the following decision options?

If possible, please provide contact information.

	Name of contact	Organization	Email address
Newborn screening of cystic fibrosis (immunoreactive trypsin test as first tier)			
Newborn screening of cystic fibrosis (single mutation DNA analysis as second tier)			
Newborn screening of cystic fibrosis (multiple mutation DNA analysis as second tier)			
Newborn screening of medium chain acyl-CoA dehydrogenase deficiency (MCADD) using tandem mass spectrometry			
Newborn screening of congenital adrenal hyperplasia (CAH) (biochemical testing methodology, measurement of 17alpha-hydroxyprogesterone)			
Newborn screening of duchenne muscular dystrophy (measurement of creatine kinase activity)			
Newborn screening of galactosaemia (photometric or fluorimetric enzyme assays)			

→ **GO TO PAGE 25.**

In the following three questions, we are interested in information about the third party payer responsible for making the decision about coverage/reimbursement.

2.	**Please indicate the country.**
	Insert name of country:

3.	**Please state the name of the primary payer for whom the decision on coverage/reimbursement was made.[2]**
	If screening was funded by more than one payer then please name the payer who provides the funds for initial screening for the condition. Please exclude and do not consider follow-up diagnosis and testing.<br>(1)<br>(2)<br>(3)
	Please insert further comments if your answer is not sufficiently covered by the options provided.

[2] **Third party payer/ payer:** A public (or private) institution that funds technologies in a healthcare system, e.g. a tax-funded payer, a statutory health insurance or a private insurance.

Example England: Newborn screening is covered by the *English National Health Service (NHS)*.
Example Sweden: HNPCC screening and genetic counselling is covered by the *Swedish National Health Service* which is organized regionally. Here *4 regions*, jointly decided to introduce the service. In other cases, it is possible that even single insurance companies reimburse a service.

4.	**Did a separate decision making institution make the decision on behalf of the third party payer?** [3]			
	Select one answer for each decision option.			
		Yes<br>*(If ticked go to question 4a)*	No<br>*(If ticked go to question 5)*	Other, please specify below<br>*(If ticked to question 4a)*
	(1)	○	○	○
	(2)	○	○	○
	(3)	○	○	○
	Please insert further comments if your answer is not sufficiently covered by the options provided.			

[3] **Decision making institution:** An institution aimed at ensuring an orderly process for decision-making to produce guidance about the use of healthcare technologies in clinical practice (e.g. the National Institute of Health and Clinical Excellence in the United Kingdom, or others).

Example England: The *National Screening Committee* which is a decision making authority of the NHS makes decisions which screening procedures to include.
Example Sweden: The four southern Swedish regions have installed a *joint administrative body (Södra Regionvårdsnämnden)*. Within this organization, the *medical council (subgroup genetic council)* decided upon introduction of the service.

ONLY ANSWER THIS QUESTION IF YOU HAVE SELECTED "YES" AS AN ANSWER IN QUESTION 4.

**4a.**	**Please state the name of the institution that made the decision for the third party payer.** [4]
	Only consider initial screening. Please exclude and do not consider follow-up diagnosis and testing.<br>(1) ☐<br>(2) ☐<br>(3) ☐
	Please insert further comments if your answer is not sufficiently covered by the options provided.

[4] **Decision making institution:** Individual or institution determining provision and funding of a medical service, e.g. the National Institute of Health and Clinical Excellence in England, a doctor or hospital manager with budget responsibility

Example England: The *National Screening Committee* which is a decision making authority of the NHS makes decisions which screening procedures to include.
Example Sweden: The four southern Swedish regions have installed a *joint administrative body (Södra Regionvårdsnämnden)*. Within this organization, the *medical council (subgroup genetic council)* decided upon introduction of the service.

5.	**Was coverage/reimbursement provided after the decision process?** [5]			
	Select one answer for each decision option.			
		Yes, full coverage	Yes, partly (not all costs are covered)	No
	(1)	○	○	○
	(2)	○	○	○
	(3)	○	○	○
	Please insert further comments if your answer is not sufficiently covered by the options provided.			

[5] **Coverage/reimbursement:** (Partial) funding of any medical service provision (e.g. genetic testing in population-based screening programs) by a third party payer being either public or private
Decision outcome: The result of a decision making process

Example England: All costs of the screening are covered by the NHS. Therefore, the decision outcome is *yes, full coverage*.
Example Sweden: *Full coverage* is provided for the covered group of individuals.
In other cases, it is possible that the service is not fully covered by third party payment and for example, individuals have to contribute co-payments in order to receive the service.

In the following questions, we would like to know the unit and amount of coverage/reimbursement.

6.	Please mark the type(s) of coverage/reimbursement AFTER the decision.[6]					
	You may select several categories if there are many funding sources (e.g. global budget for laboratory and an additional fee per test).					
	Per test (fee for service)	Per insured (capitation based payment)	Per year (annual budget)	Per diagnosis related group (DRG)-based case	Co-payment per insured	Other, please specify below
(1)	☐	☐	☐	☐	☐	☐
(2)	☐	☐	☐	☐	☐	☐
(3)	☐	☐	☐	☐	☐	☐
	Please insert further comments if your answer is not sufficiently covered by the options provided.					

[6] **Coverage/reimbursement:** (Partial) funding of any medical service provision (e.g. genetic testing in population-based screening programs) by a third party payer being either public or private

Example England and Sweden: In both cases, the screenings are funded from *a fixed annual budget*.
In other cases, it is possible that other reimbursement mechanisms apply. E.g. if the screening service is not organized within centralized structures, clinics and laboratories might receive a fee per test, or, the service is reimbursed per insured individual. If the service is usually provided in a clinic, it is possible that the service is provided within a diagnosis related group.

7.	**Please mark the type(s) of coverage/reimbursement BEFORE the decision.**[7]						
	You may select several categories if there are many funding sources (e.g. global budget for laboratory and an additional fee per test).						
		Per test (fee for service)	Per insured (capitation based payment)	Per year (annual budget)	Per diagnosis related group (DRG)-based case	Co-payment per insured	Other, please specify below
	(1)	☐	☐	☐	☐	☐	☐
	(2)	☐	☐	☐	☐	☐	☐
	(3)	☐	☐	☐	☐	☐	☐
	Please insert further comments if your answer is not sufficiently covered by the options provided.						

[7] **Coverage/reimbursement:** (Partial) funding of any medical service provision (e.g. genetic testing in population-based screening programs) by a third party payer being either public or private

Example England and Sweden: In both cases, the screenings are funded from *a fixed annual budget*.
In other cases, it is possible that other reimbursement mechanisms apply. E.g. if the screening service is not organized within centralized structures, clinics and laboratories might receive a fee per test, or, the service is reimbursed per insured individual. If the service is usually provided in a clinic, it is possible that the service is provided within a diagnosis related group.

8.	**Please specify, if a separate amount of coverage/reimbursement can be identified for the decision option.** [8]
	If the amount of reimbursement cannot be stated separately for each decision option (e.g. it is included in a budget for several services or there is a mix of funding), please specify in the commenting field. If of no coverage/reimbursement or co-payment is provided, please type "0". Only consider the amount of coverage/reimbursement after the decision was made.

	Amount of coverage/reimbursement (including supplementary services, e.g. genetic testing with counselling)	Co-payment by insured individual	Funding from other sources (e.g. average reimbursement from private insurance), please specify below
(1)			
(2)			
(3)			

Please insert further comments if your answer is not sufficiently covered by the options provided.

9.	**Please indicate the currency for coverage/reimbursement.**

Select one answer.

- ◯ Euro
- ◯ British Pound
- ◯ Bulgarian Lev
- ◯ Czech Koruna
- ◯ Danish Krone
- ◯ Estonian Kroon
- ◯ Hungarian Forint
- ◯ Latvian Lats
- ◯ Lithuanian Litas
- ◯ Polish Zloty
- ◯ Romanian Leu
- ◯ Swedish Krona
- ◯ Other, please specify: ____

[8] **Example England:** For the newborn screening of sickle cell diseases *159,240* British Pound were spent for screening alone. For the programme development and a programme centre which also includes counselling and antenatal screening, the total budget amounted to 1,981,444 British Pound. Since this is a global budget for all services, the separate costs of sickle cell newborn screening cannot be identified. No co-payments or funding from other sources exist.
Example Sweden: The total annual budget for screening and family counselling for genetic disorders amounts to 9.226.800 Swedish Kronor in total in 2008. However, no separate budget can be identified for HNPCC screening alone. No co-payments or funding from other sources exist

In the following question, we would like you to think about the level of service provision. We would like you to think about this (a) before the final coverage/ reimbursement decision was made and (b) a reasonable time after the decision was made and the service was implemented. This is because a decision may have been reached to exclude a service from coverage/reimbursement which was available previously.
If the decision to provide the service is made on an individual patient basis (case-by-case) then use the time point when the general criteria of entitlement to the service was decided.

10. Please indicate, in your view, the level of service provision of the screening/ diagnostic service (a) before the coverage/ reimbursement decision and (b) after the service was implemented. [9]

Appropriate provision: The payer provides the screening/ diagnostic service for all patients that meet the entitlement criteria.
Under-provision: The payer provides the screening/ diagnostic service for fewer patients than meet the entitlement criteria.
Over-provision: The payer provides the screening/ diagnostic service for more patients than meet the entitlement criteria.

Select one answer for each decision option.

	(a) Service provision BEFORE decision	(b) Service provision AFTER service was implemented
(1)	◯ Appropriate service provision<br>◯ Underprovision<br>◯ Overprovision<br>◯ Both under- and overprovision<br>◯ No service provision	◯ Appropriate service provision<br>◯ Underprovision<br>◯ Overprovision<br>◯ Both under- and overprovision<br>◯ No service provision
(2)	◯ Appropriate service provision<br>◯ Underprovision<br>◯ Overprovision<br>◯ Both under- and overprovision<br>◯ No service provision	◯ Appropriate service provision<br>◯ Underprovision<br>◯ Overprovision<br>◯ Both under- and overprovision<br>◯ No service provision
(3)	◯ Appropriate service provision<br>◯ Underprovision<br>◯ Overprovision<br>◯ Both under- and overprovision<br>◯ No service provision	◯ Appropriate service provision<br>◯ Underprovision<br>◯ Overprovision<br>◯ Both under- and overprovision<br>◯ No service provision

Please insert further comments if your answer is not sufficiently covered by the options provided.

[9] **Service provision:** Mode of how medical services are offered to the covered individuals

Example England: The programme was fully implemented within the first year after the final decision, leading to *appropriate service provision*. Before, only those newborns received screening which were born in the areas where pilot studies were conducted. However at this time, there was no positive decision to fund sickle cell screening which means that there was *overprovision* of the service, since it was offered to a part of the covered population.
Example Sweden: No information was available.
It is possible that certain groups of patients that are eligible to receive the service do not receive it while others which are not eligible to receive the service receive it leading to both under- and overprovision. In other cases, the service was neither offered nor funded at all before a decision was made. However from the viewpoint before the decision, this was considered as appropriate service provision.

In the following question, we would like to understand whether, and to what extent, the payer has implemented a policy to monitor service provision (e.g. policies of pre-authorization, detailed quality reporting).

11. Which information has to be reported from the service provider to the payer? [10]

Several answers are possible for each decision option. Do not consider any clinical follow-up of screened/diagnosed individuals.

	Number of reimbursed services	Specific information about services	Pre-authorization required	No additional information required	Other, please specify below
(1)	☐	☐	☐	☐	☐
(2)	☐	☐	☐	☐	☐
(3)	☐	☐	☐	☐	☐

Please insert further comments if your answer is not sufficiently covered by the options provided.

[10] **Service provision:** Mode of how medical services are offered to the covered individuals

Example England: The Primary Care Trusts which offer the service need to report the *number of reimbursed services* as well as additional data *(specific information about services)*.
Example Sweden: No information was available.
In other cases it is possible, that a service may only by provided after pre-authorization, e.g. if the patient fulfils certain criteria to receive the service which the service provider needs to record.

In this question, we want to understand the differences between decisions made for a large group of individuals compared to decisions made only for a few individuals.

12. How many screenings/diagnostic services are expected to be covered/ reimbursed? [11]

Please insert the number of expected screenings/diagnostic services in the left column. A rough estimate is sufficient. For screening, the number of screened individuals (not detected individuals) is meant. The total number is the number / interval (multiplied by the number of intervals if time horizon restricted).

	Number of screenings/ diagnostic services	Please specify interval (e.g. per year, per month, per day) or write "in total"	Please specify the date until the decision is valid or write "unrestricted time horizon"
(1)			
(2)			
(3)			

Please insert further comments if your answer is not sufficiently covered by the options provided.

[11] **Example England:** From April 2005 to March 2007 1.1 Mio newborns were screened in total in England for sickle cell diseases. This corresponds with approximately *560,000* screened infants per year and the expected number of reimbursed screenings.

Example Sweden: In 1997, *220* patients received screening and counselling in the respective region. This corresponds with the expected number of reimbursed services.

In the following five questions we would like to find out more about the decision process.

In the following question, please state how the decision process was initiated. We would like to know if a formalized process for selecting decision options exists. This information is important because it helps us to understand why some other technologies have not undergone a formal decision and if so, what kind of processes exist.

13. **How was the decision process started?**[12]

Select one answer for each decision option.

	Ad-hoc selection of decision option	Explicit specification of criteria for start of decision process (e.g. budget impact, unmet need)	Other, please specify below
(1)	○	○	○
(2)	○	○	○
(3)	○	○	○

Please insert further comments if your answer is not sufficiently covered by the options provided.

[12] **Formal:** Any kind of activity within the decision making process that has been regulated according to any kind of regulation, e.g. by a statute or guideline

Example England: The decision procedure is started by initiative and expert opinion of the members of the National Screening Committee. However, the appraisal criteria might be applied in the first stages of the decision process as well *(Explicit specification of criteria for start of decision process)*.
Example Sweden: Clinicians of the region took initiative to introduce HNPCC counselling and screening. No formal procedure exists to start the decision process *(Ad-hoc selection)*.

Stakeholder involvement plays an important role in decision making. In the following two questions, we would like to find out who was involved in the decision process and the extent and level of the stakeholders' involvement.

14. Which types of stakeholders were formally involved in the decision process?[13]

This question only addresses formal stakeholder participation as specified by law or similar statutes which can be for example information provision, appealing to the decision or participation in the vote on the outcome of the decision process. Informal participation such as lobbying activities is not addressed here.

	Service provi-der(s)	Payer	Govern-ment	Patients / patient represen-tatives	Industry	Aca-demia	Health tech-nology assess-ment group or agency	Others, please specify below
(1)	☐	☐	☐	☐	☐	☐	☐	☐
(2)	☐	☐	☐	☐	☐	☐	☐	☐
(3)	☐	☐	☐	☐	☐	☐	☐	☐

Please insert further comments if your answer is not sufficiently covered by the options provided.

[13] **Stakeholder:** Any organization or individual (group of individuals) with the intention to participate at any step/stage of the decision making procedure having any kind of interest in the decision
Formal: Any kind of activity within the decision making process that has been regulated according to any kind of official regulation, e.g. by law, a guideline of the decision making process of the payer/decision making institution

Example England: A formal decision process exists for inclusion of new screening procedures. The NHS *(payer)* was involved. The health technology assessments were conducted by a collaborative group from different universities *(health technology assessment group or agency, academia)*. Implementation and support of the pilot studies was provided by the Sickle Cell and Thalassaemia Programme *(service providers, academia)*. It is possible that in other cases, the respective Ministry of Health *(government)*, manufacturers of analyzing specimens *(industry)* or patient organizations *(patients)* participated as stakeholders in the process as well.
Example Sweden: In the decision on HNPCC screening and counselling, doctors *(service providers)* and *academia* as well as the *payer* represented by the medical council were involved in the decision making. However, no formal decision process exists on this level of decision making.

ONLY CONSIDER THOSE STAKEHOLDERS THAT YOU HAVE SELECTED IN QUESTION 14.

15. **What was the level of involvement of the selected stakeholder?** [14]

Several answers are possible per decision option and stakeholder. Informal participation like lobbying activities is not addressed here. Please note that due to technical circumstances the question is displayed differently in the online version of the questionnaire.

	Service provider(s)	Payer	Government	Patients / patient representatives	Industry	Academia	Health technology assessment group or agency	Others, please specify below
(1)	☐ Information provision<br>☐ Appeal<br>☐ Vote<br>☐ Other, please specify below	☐ Information provision<br>☐ Appeal<br>☐ Vote<br>☐ Other, please specify below	☐ Information provision<br>☐ Appeal<br>☐ Vote<br>☐ Other, please specify below	☐ Information provision<br>☐ Appeal<br>☐ Vote<br>☐ Other, please specify below	☐ Information provision<br>☐ Appeal<br>☐ Vote<br>☐ Other, please specify below	☐ Information provision<br>☐ Appeal<br>☐ Vote<br>☐ Other, please specify below	☐ Information provision<br>☐ Appeal<br>☐ Vote<br>☐ Other, please specify below	☐ Information provision<br>☐ Appeal<br>☐ Vote<br>☐ Other, please specify below
(2)	☐ Information provision<br>☐ Appeal<br>☐ Vote<br>☐ Other, please specify below	☐ Information provision<br>☐ Appeal<br>☐ Vote<br>☐ Other, please specify below	☐ Information provision<br>☐ Appeal<br>☐ Vote<br>☐ Other, please specify below	☐ Information provision<br>☐ Appeal<br>☐ Vote<br>☐ Other, please specify below	☐ Information provision<br>☐ Appeal<br>☐ Vote<br>☐ Other, please specify below	☐ Information provision<br>☐ Appeal<br>☐ Vote<br>☐ Other, please specify below	☐ Information provision<br>☐ Appeal<br>☐ Vote<br>☐ Other, please specify below	☐ Information provision<br>☐ Appeal<br>☐ Vote<br>☐ Other, please specify below
(3)	☐ Information provision<br>☐ Appeal<br>☐ Vote<br>☐ Other, please specify below	☐ Information provision<br>☐ Appeal<br>☐ Vote<br>☐ Other, please specify below	☐ Information provision<br>☐ Appeal<br>☐ Vote<br>☐ Other, please specify below	☐ Information provision<br>☐ Appeal<br>☐ Vote<br>☐ Other, please specify below	☐ Information provision<br>☐ Appeal<br>☐ Vote<br>☐ Other, please specify below	☐ Information provision<br>☐ Appeal<br>☐ Vote<br>☐ Other, please specify below	☐ Information provision<br>☐ Appeal<br>☐ Vote<br>☐ Other, please specify below	☐ Information provision<br>☐ Appeal<br>☐ Vote<br>☐ Other, please specify below

Please insert further comments if your answer is not sufficiently covered by the options provided.

[14] **Appeal:** The official right of an involved stakeholder or individual to raise objections against the decision process or the (planned) outcome of the decision
Stakeholder: Any organization or individual (group of individuals) with the intention to participate at any step/stage of the decision making procedure having any kind of interest in the decision
Vote: The official right of a stakeholder to participate in the final decision making having own voting rights

Example England: Presumably, the people who conducted the health technology assessment and patient representatives *provided information*. Here, no stakeholder made an appeal during or after the decision process.
Example Sweden: No information available

In this question, we are interested in the degree of transparency to external individuals and institutions. This is assessed in terms of the availability of publicly accessible information.

16. Which information was accessible by the public DURING the decision process? [15]

Several answers are possible for each decision option.

	Attendance at or minutes of appraisal meeting	Decision rationale	Decision outcome	Health technology assessment report	Stake-holder comments	Rationale for assess-ment question from scoping	No infor-mation available	Other, please specify below
(1)	☐	☐	☐	☐	☐	☐	☐	☐
(2)	☐	☐	☐	☐	☐	☐	☐	☐
(3)	☐	☐	☐	☐	☐	☐	☐	☐

Please insert further comments if your answer is not sufficiently covered by the options provided.

[15] **Publicly accessible:** Documentation of the decision making process open to all individuals covered by the services of the payer via internet or print publication or other sources (e.g. telephone hotline)
Scoping: Stage during decision process where exact research question for assessment is defined (e.g. in a workshop with all participating stakeholders)

Example England: The minutes of the National Screening Committee *(Appraisal meeting)* are available as well as health technology assessments and other related documents *(decision rationale and outcome, further supporting information)*.
Example Sweden: The *decision rationale and outcome* were available as well as *further supporting information (annual budget information)*. There is no information on assessment.

17. Which information was accessible by the public AFTER the decision process?[16]

Several answers are possible for each decision option.

	Attendance at or minutes of appraisal meeting	Decision rationale	Decision outcome	Health technology assessment report	Stake-holder comments	Rationale for assess-ment question from scoping	No infor-mation available	Other, please specify below
(1)	☐	☐	☐	☐	☐	☐	☐	☐
(2)	☐	☐	☐	☐	☐	☐	☐	☐
(3)	☐	☐	☐	☐	☐	☐	☐	☐

Please insert further comments if your answer is not sufficiently covered by the options provided.

[16] **Publicly accessible:** Documentation of the decision making process open to all individuals covered by the services of the payer via internet or print publication or other sources (e.g. telephone hotline)
Scoping: Stage during decision process where exact research question for assessment is defined (e.g. in a workshop with all participating stakeholders)

Example England: The minutes of the National Screening Committee *(Appraisal meeting)* are available as well as health technology assessments and other related documents *(decision rationale and outcome, further supporting information)*.
Example Sweden: The *decision rationale and outcome* were available as well as *further supporting information (annual budget information)*. There is no information on assessment.

In the following four questions we are interested in understanding the criteria that guide the decision process.

In this question, we would like to understand if and, how the evidence of effectiveness of the technology was assessed.

18. How was evidence assessed during the decision process?[17]

More than one answer is possible for each decision option. In case assessment methods specific for genetics (e.g. ACCE, EGAPP) or diagnosis / screening (e.g. ROC curves, Wilson & Jungner Criteria) were used, please specify briefly below

	No assessment of effectiveness	Expert opinion	Systematic literature review	Quantitative meta-analysis of studies	Other, please specify below
(1)	☐	☐	☐	☐	☐
(2)	☐	☐	☐	☐	☐
(3)	☐	☐	☐	☐	☐

Please insert further comments if your answer is not sufficiently covered by the options provided.

[17] **Evidence of effectiveness:** Degree of quality of information regarding the efficacy as well as effectiveness available for a technology under evaluation

Example England: Evidence of effectiveness of screening of sickle cell diseases was assessed by systematic search of the literature as part of a health technology assessment *(systematic literature review)*.
Example Sweden: For the decision on HNPCC screening and counselling no explicit assessment on effectiveness was conducted. However, participating clinicians provided information on effectiveness *(expert opinion)*.

In this question, we would like to know whether cost or cost-effectiveness data was used during the decision process.

19. If costs or cost-effectiveness data was used in the decision process, what information was this assessment based on at least? [18]

Select one answer for each decision option.

	No assessment of costs	Rough estimate of costs	Formalized cost estimate	Cost-effectiveness analyses *without* consideration of pre-specified guidelines	Cost-effectiveness analyses *fully in line with* pre-specified guidelines
(1)	○	○	○	○	○
(2)	○	○	○	○	○
(3)	○	○	○	○	○

Please insert further comments if your answer is not sufficiently covered by the options provided.

[18] **Cost-effectiveness:** Comparison of costs of one or more technologies in relation to the effects related with the technology (-ies). Effects can be measured using clinical parameters (cost-effectiveness analysis), health related quality of life (cost-utility analysis) or monetary units (cost-benefit analysis).

Example England: As part of the health technology assessment, the research group assessed the cost-effectiveness by estimating the costs per extra case identified through the programme. However, the assessment was not conducted according to pre-specified criteria *(Cost-effectiveness analyses in general)*. In other assessments, costs per quality adjusted life year (QALY) or other measures might be assessed.
Example Sweden: Costs or cost-effectiveness were not explicitly assessed by the involved clinicians *(No assessment of costs)*.

In the following two questions, we would like to know about the influence of certain aspects on the outcome of the decision making process.

20. Which of the following aspects mentioned below were relevant for the decision on coverage/reimbursement? Please TICK all relevant aspects.

More than one answer is possible for each decision option.

21. From the aspects you have selected, please CIRCLE those aspects which, in your view, had the highest impact on the outcome of the decision process.

More than one answer is possible for each decision option. Please note that due to technical circumstances the question is displayed differently in the online version of the questionnaire.

(1)

Influences from assessment	External influences
☐ Effectiveness (health gain from testing)	☐ Lobbying activities by service provider(s)
☐ Effectiveness (other benefit of knowledge from testing)	☐ Lobbying activities by industry
☐ Expected costs	☐ Lobbying activities by patients/ patient representatives
☐ Cost-effectiveness	☐ Lobbying by government
☐ Budget impact	☐ Third party payer's concern for cost containment
☐ Effect on equitable access to health care	☐ Other, please specify below
☐ Severity of the disease	
☐ Quality of evidence	
☐ Availability of treatment for disease	
☐ Scientific interest in gathering further evidence	

(2)

Influences from assessment	External influences
☐ Effectiveness (health gain from testing)	☐ Lobbying activities by service provider(s)
☐ Effectiveness (other benefit of knowledge from testing)	☐ Lobbying activities by industry
☐ Expected costs	☐ Lobbying activities by patients/ patient representatives
☐ Cost-effectiveness	☐ Lobbying by government
☐ Budget impact	☐ Third party payer's concern for cost containment
☐ Effect on equitable access to health care	☐ Other, please specify below
☐ Severity of the disease	
☐ Quality of evidence	
☐ Availability of treatment for disease	
☐ Scientific interest in gathering further evidence	

(3)

Influences from assessment	External influences
☐ Effectiveness (health gain from testing)	☐ Lobbying activities by service provider(s)
☐ Effectiveness (other benefit of knowledge from testing)	☐ Lobbying activities by industry
☐ Expected costs	☐ Lobbying activities by patients/ patient representatives
☐ Cost-effectiveness	☐ Lobbying by government
☐ Budget impact	☐ Third party payer's concern for cost containment
☐ Effect on equitable access to health care	☐ Other, please specify below
☐ Severity of the disease	
☐ Quality of evidence	
☐ Availability of treatment for disease	
☐ Scientific interest in gathering further evidence	

Please insert further comments if your answer is not sufficiently covered by the options provided.

22. What was the level of your involvement in the decision process?

If you are answering the survey on behalf of someone else we would like to know **your** role. Select one category for each decision option.

	Direct involvement in decision process	Well informed observer without direct involvement	Expert without relation to decision
(1)	◯	◯	◯
(2)	◯	◯	◯
(3)	◯	◯	◯

Please insert further comments if your answer is not sufficiently covered by the options provided.

23. Are you aware of any other person who might be able to provide information on the decision options?

If possible, please provide contact information.

	Name of contact	Organization	Email address
(1)			
(2)			
(3)			

Please insert further comments if your answer is not sufficiently covered by the options provided.

At the end of this survey, we have some general questions.

**Please indicate your name and institution.**	
First name	
Last name	
Institution	

**Do you want your name be kept confidential?**	
○ Yes	○ No

**Do you want your institution be kept confidential?**	
○ Yes	○ No

**May we contact you to invite you to take part in further research regarding this project?**	
○ Yes, you may contact me. Please use this email address: ______________	○ No, please do not contact me any further

**If you are interested in a summary of the results, please provide your email address here.**

**In case you have comments on this questionnaire, we are grateful about your feedback!**

Thank you very much for participating in our survey on coverage and reimbursement decisions!

For questions or comments do not hesitate to contact:

Helmholtz Zentrum Muenchen
German Research Center for Environmental Health
Institute for Health Economics and Health Care Management
Director: Prof. Dr. Reiner Leidl

Website: http://www.helmholtz-muenchen.de/igm
Fax: +49 89 3187-3375

Diplom-Volkswirtin Katharina Fischer (Economist)
Email: katharina.fischer@helmholtz-muenchen.de
Telephone: +49 89 3187-4157

Dr. Wolf Rogowski
Email: rogowski@helmholtz-muenchen.de

Please return this questionnaire by mail to:
Helmholtz Zentrum Muenchen
Institute of Health Economics and Health Care Management
Katharina Fischer
Ingolstaedter Landstr. 1
85764 Neuherberg/Germany

Or fax to:
+49 89 3187-3375

Email template for invitation to web-based questionnaire

Customized text in *italics*.

Dear *name of respondent*

Thank you very much for participating in our survey on decision making about health care resources! Your expertise is essential in understanding how decisions on (newborn) screening technologies are made in your country. This survey is part of a project funded by the European Union (HIScreenDiag) and focuses on the description and analysis of decision processes of screening and diagnostic technologies with a special focus on genetic testing.

HIScreenDiag is funded by the EU under Framework Seven (grant no. 223533, for more information see attachment). It is a collaborative project of the following universities and publicly funded research centers:

- Helmholtz Center Munich (Germany)
- Institute of Prospective Technology Studies (Sevilla, Spain)
- University of La Rioja (Spain)
- University of Gent (Belgium)
- Universtiy of Groningen (Netherlands)
- University of Manchester (United Kingdom)

The survey should take no more than 30 minutes to complete. The majority of the questions are structured but welcome your comments and any additional information in the space provided.

Access to the survey is protected. Please use following information for login:

Login: *email address*

Password: *password*

Please click here to begin: https://ww3.unipark.de/uc/HIScreenDiag

(If the secured link does not work please use the following: http://ww3.unipark.de/uc/HIScreenDiag)

If you prefer, you may take this as a telephone based survey. If you do not want to take the survey online, the questionnaire is attached for printout.

Feel free to contact us in case you have questions or problems starting the online survey: katharina.fischer@helmholtz-muenchen.de, or telephone: +49 89 3187-4157.

Again, thank you so much for your cooperation! If you specify at the end of the questionnaire, we are delighted to send you a summary of the survey results.

Kind regards

Katharina Fischer on behalf of the research team
