# Supplementary material for: Decision-making in healthcare: a practical application of partial least square path modelling to coverage of newborn screening programmes
Source: BMC Med Inform Decis Mak. 2012 Aug 2;12:83. doi: 10.1186/1472-6947-12-83 (PMC3444310; doi:10.1186/1472-6947-12-83)
Supplement: Additional file 3 — Evaluation measures. [file 1472-6947-12-83-S3.doc]

# Decision-making in health care: A practical application of partial least square path modelling to coverage of newborn screening programs

## Additional file 3: Evaluation measures for assessment of SEM for coverage decision-making

### 1. Original model, reflective measurement models

Table I − Construct reliability and convergent validity

| **Construct** | **Composite Reliability** | **Average Variance Extracted** |
| --- | --- | --- |
| Participation | 0.6059 | 0.4291 |
| Reasonableness | 0.6703 | 0.2691 |
| Scientific rigour of assessment | 0.6721 | 0.5495 |
| Transparency | 0.9234 | 0.8578 |

Table II − Indicator loadings for reflective measurement models

| **Reasonableness** | | | **Transparency** | |
| --- | --- | --- | --- | --- |
| appr_BI | | 0.3930 | info_number | 0.8957 |
| appr_CE | | 0.4399 | transparency | 0.9557 |
| appr_access | | 0.4334 |  |  |
| appr_effect1 | | 0.7109 |  |  |
| appr_effect2 | | 0.3240 |  |  |
| appr_sever | | 0.6846 |  |  |
| **Participation** | | | **Scientific rigour of assessment** | |
| involvement_appeal | -0.2597 | | assess_cost | 0.3830 |
| involvement_info | 0.8133 | | assess_effect | 0.9759 |
| involvement_voting | 0.4187 | |  |  |
| part_number | 0.9011 | |  |  |

Table III − Determinant validity: Fornell−Larcker criterion

| **Construct**  **Construct** | Participation | Reasonable­ness | Scientific rigour of assessment | Transparency |
| --- | --- | --- | --- | --- |
| Participation | 0.4291 |  |  |  |
| Reasonableness | 0.3681 | 0.2691 |  |  |
| Scientific rigour of assessment | 0.0011 | 0.0346 | 0.5495 |  |
| Transparency | 0.1303 | 0.2143 | 0.0624 | 0.8578 |

Table IV − Determinant validity: cross-loadings

| **Construct**  **Indicator^a^** | Participation | Reasonable­ness | Scientific rigour of assessment | Transparency |
| --- | --- | --- | --- | --- |
| appr_BI | **0.2765** | 0.3930 | -0.1459 | 0.1107 |
| appr_CE | **0.2402** | 0.4399 | 0.2377 | 0.2101 |
| appr_access | **0.0434** | 0.4334 | -0.0718 | 0.1873 |
| appr_effect1 | **0.4453** | 0.7109 | 0.1688 | 0.5013 |
| appr_effect2 | **0.2727** | 0.3240 | 0.0811 | 0.1646 |
| appr_sever | **0.3861** | 0.6846 | 0.1016 | 0.0670 |
| assess_cost | **0.0967** | 0.1299 | 0.3830 | 0.0230 |
| assess_effect | **0.0119** | 0.1676 | 0.9759 | 0.2609 |
| info_number | 0.1595 | 0.2943 | 0.2442 | **0.8957** |
| involvement_appeal | -0.2597 | -0.0973 | -0.3618 | 0.1268 |
| involvement_info | 0.8133 | **0.4862** | 0.1541 | 0.3812 |
| involvement_voting | 0.4187 | **0.1913** | -0.2418 | 0.0126 |
| part_number | 0.9011 | **0.5843** | -0.0718 | 0.3376 |
| transparency | 0.4534 | 0.5221 | 0.2251 | **0.9557** |
| ^a^ Indicators that define construct in grey. Indicators at maximum in bold. | | | | |

### 2. SEM model selection, measurement models and structural model

Table V − Construct reliability, convergent validity and structural model

| **Measure**  **Construct** | **Reflective measurement models** | | **Structural model** | |
| --- | --- | --- | --- | --- |
|  | Composite Reliability | Average Variance Extracted | Determination coefficient R^2^ | Stone-Geisser test Q^2^ |
| Participation | 0.7556 | 0.5411 | - | - |
| Reasonableness | 0.7166 | 0.3423 | 0.3811 | 0.0405 |
| Scientific rigour of assessment | 0.6588 | 0.5434 | 0.0698 | -0.0105 |
| Transparency | 0.9229 | 0.8571 | - | - |

Table VI − Indicator loadings

| **Reasonableness** | | **Transparency** | |
| --- | --- | --- | --- |
| appr_BI | 0.5231 | info_number | 0.8930 |
| appr_CE | 0.5027 | transparency | 0.9574 |
| appr_access | 0.4723 |  |  |
| appr_effect1 | 0.6589 |  |  |
| appr_sever | 0.7266 |  |  |
| **Participation** | | **Scientific rigour of assessment** | |
| involvement_info | 0.8753 | assess_cost | 0.3437 |
| involvement_voting | 0.3181 | assess_effect | 0.9842 |
| part_number | 0.8694 |  |  |

Table VII − Determinant validity: Fornell−Larcker criterion

| **Construct**  **Construct** | Participation | Reasonable­ness | Scientific rigour of assessment | Trans­parency |
| --- | --- | --- | --- | --- |
| Participation | 0.5411 |  |  |  |
| Reasonable­ness | 0.3282 | 0.3423 |  |  |
| Scientific rigour of assessment | 0.0010 | 0.0247 | 0.5434 |  |
| Transparency | 0.1646 | 0.1827 | 0.0638 | 0.8571 |

Table VIII − Determinant validity: cross-loadings

| **Construct**  **Indicator** | **Participation** | **Reasonable­ness** | **Scientific rigour of assessment** | **Trans­parency** |
| --- | --- | --- | --- | --- |
| appr_BI | 0.3216 | 0.5231 | -0.1553 | 0.1130 |
| appr_CE | 0.2824 | 0.5027 | 0.2233 | 0.2109 |
| appr_access | 0.0819 | 0.4723 | -0.0714 | 0.1863 |
| appr_effect1 | 0.4261 | 0.6589 | 0.1786 | 0.5022 |
| appr_sever | 0.3938 | 0.7266 | 0.0974 | 0.0678 |
| assess_cost | 0.1131 | 0.1357 | 0.3437 | 0.0219 |
| assess_effect | 0.012 | 0.1395 | 0.9842 | 0.2608 |
| info_number | 0.2299 | 0.2573 | 0.2434 | 0.8930 |
| involvement_info | 0.8753 | 0.5410 | 0.1465 | 0.3820 |
| involvement_voting | 0.3181 | 0.0901 | -0.2505 | 0.0139 |
| part_number | 0.8694 | 0.4663 | -0.0704 | 0.3398 |
| transparency | 0.4734 | 0.4890 | 0.2304 | 0.9574 |

Table IX − t-statistics from bootstrapping procedure

| **Measure** | **t-statistic** | **p value** |
| --- | --- | --- |
| *Outer loadings* |  |  |
| appr_BI ← Reasonableness | 2.2327 | 0.02974 |
| appr_CE ← Reasonableness | 2.4055 | 0.01961 |
| appr_access ← Reasonableness | 2.5794 | 0.01265 |
| appr_effect1 ← Reasonableness | 2.9107 | 0.00523 |
| appr_sever ← Reasonableness | 4.6297 | 0.00002 |
| assess_cost ← Scientific rigour of assessment | 1.175 | 0.24515 |
| assess_effect ← Scientific rigour of assessment | 3.4453 | 0.00111 |
| info_number ← Transparency | 10.2666 | 0.00000 |
| involvement_info ← Participation | 5.5487 | 0.00000 |
| involvement_voting ← Participation | 1.3909 | 0.16996 |
| part_number ← Participation | 5.6712 | 0.00000 |
| transparency ← Transparency | 11.7554 | 0.00000 |
| *Path coefficients* |  |  |
| Participation → Reasonableness | 3.8154 | 0.00035 |
| Participation → Scientific rigour of assessment | 0.5574 | 0.57956 |
| Scientific rigour of assessment → Reasonableness | 0.5721 | 0.56963 |
| Transparency → Reasonableness | 1.6829 | 0.09817 |
| Transparency → Scientific rigour of assessment | 1.9953 | 0.05107 |

Table X − Exogenous constructs’ contribution to endogenous constructs

| **Effect size f^2^**  **Exogenous constructs** | Scientific rigour of assessment | Reasonableness |
| --- | --- | --- |
| Participation | 0.0052 | 0.1811 |
| Transparency | 0.0578 | 0.0454 |
